# Brain-computer-interface-based intervention increases brain functional segregation in cognitively normal older adults

**DOI:** 10.1093/ageing/afaf250

**Published:** 2025-09-12

**Authors:** Xing Qian, Kwun Kei Ng, Si Ning Yeo, Yng Miin Loke, Yin Bun Cheung, Lei Feng, Mei Sian Chong, Tze Pin Ng, K Ranga Rama Krishnan, Cuntai Guan, Tih-Shih Lee, Juan Helen Zhou

**Affiliations:** Centre for Sleep and Cognition and Centre for Translational Magnetic Resonance Research, Yong Loo Lin School of Medicine, National University of Singapore, Singapore, Singapore; Centre for Sleep and Cognition and Centre for Translational Magnetic Resonance Research, Yong Loo Lin School of Medicine, National University of Singapore, Singapore, Singapore; Neuroscience and Behavioural Disorders Programme, Duke-NUS Medical School, Singapore, Singapore; Centre for Sleep and Cognition and Centre for Translational Magnetic Resonance Research, Yong Loo Lin School of Medicine, National University of Singapore, Singapore, Singapore; Centre for Quantitative Medicine, Duke-NUS Medical School, Singapore, Singapore; Department of Psychological Medicine, Yong Loo Lin School of Medicine, National University of Singapore, Singapore, Singapore; Geriatric Education and Research Institute, Ministry of Health, Singapore, Singapore; Department of Psychological Medicine, Yong Loo Lin School of Medicine, National University of Singapore, Singapore, Singapore; Department of Psychiatry, Rush Medical College, Chicago, IL, USA; College of Computing and Data Science, Nanyang Technological University, Singapore, Singapore; Neuroscience and Behavioral Disorders Programme, Duke-NUS Medical School, Singapore, Singapore; Centre for Sleep and Cognition and Centre for Translational Magnetic Resonance Research, Yong Loo Lin School of Medicine, National University of Singapore, Singapore, Singapore; Department of Medicine, Yong Loo Lin School of Medicine, National University of Singapore, Singapore, Singapore; Department of Electrical and Computer Engineering, National University of Singapore, Singapore, Singapore; Integrative Sciences and Engineering Programme (ISEP), National University of Singapore, Singapore, Singapore

**Keywords:** fMRI, brain-computer-interface-based intervention, network, functional connectivity, ageing, older people

## Abstract

Brain-computer interface (BCI)-based cognitive training systems have shown promise in enhancing cognitive performance in cognitively normal older adults. However, the brain network changes underlying these behavioural improvements remain poorly understood. To address this gap, we investigated topological alterations in intrinsic brain functional networks following BCI-based training and their behavioural relevance in cognitively normal older adults using resting-state functional magnetic resonance imaging and graph theoretical analysis. Compared to a non-intervention waitlist (WL) group, the intervention (INT) group did not show significant behavioural improvements. However, they exhibited positive changes in brain network organisation. Specifically, the INT group demonstrated a reduced nodal participation coefficient, indicating enhanced strength of a node’s connections within its community, primarily within control and subcortical networks, as well as increased system segregation after training. Additionally, the modular organisation of the brain functional network in the INT group became more segregated and more aligned with a young adult-based partition template (quantified using the adjusted Rand index) compared to the WL group. Importantly, decreased participation coefficients, particularly in subcortical regions, were associated with language improvement, while increases in the adjusted Rand index were linked to enhancements in everyday memory function. These findings suggest that BCI-based cognitive training may contribute to maintaining brain network organisation in cognitively normal ageing by enhancing functional network segregation, potentially supporting cognitive performance. This study provides insights into the neural mechanisms underlying the effectiveness of BCI-based cognitive training for cognitively normal ageing.

## Key points

- Brain functional network segregation was improved in cognitively normal older adults, following a BCI-based cognitive intervention (INT).
- The brain’s functional modular organisation became more similar to the pattern of young healthy adults after the INT.
- These changes in functional network organisation were related to cognitive performance changes in cognitively normal older adults.

## Introduction

Population ageing is one of the most significant societal trends today [1]. Normal ageing is associated with declines in cognitive abilities, including processing speed, memory, language, visuospatial function and executive function, which can adversely affect everyday functioning and quality of life in older adults [2]. Over the past decades, researchers have increasingly focused on non-pharmacological cognitive training approaches to preserve cognitive vitality in the ageing population [3, 4].

Electroencephalography (EEG)-based neurofeedback systems, which modulate neural activity in real-time to enhance brain function, have shown promise in improving cognitive performance in healthy older adults [5–7]. A few studies hypothesised these systems could induce training-specific neural plasticity and behavioural improvements through targeted EEG signals [8–10]. A personalised brain-computer interface (BCI)-based neurofeedback system was designed to train attention and working memory in healthy older adults [5]. This system engages users in cognitive training games where feedback reflects their measured concentration levels, utilising virtual scenarios to enhance real-life transferability and sustain motivation for long-term participation. This BCI-based training has demonstrated cognitive performance gains, particularly in delayed memory and language in males, suggesting its potential as a cognitive training tool for healthy ageing. These improvements are believed to arise from training-induced neural plasticity [9, 11].

Non-invasive neuroimaging methods, such as resting-state functional magnetic resonance imaging (rs-fMRI), have opened new avenues to characterise the brain plasticity at macro level using large-scale functional networks and provided insights into the mechanisms underlying cognitive decline and enhancement. Resting-state functional connectivity (FC), which measures the synchronisation of low frequency blood-oxygenation-level-dependent (BOLD) signal fluctuations between brain regions under task-free conditions [12], has consistently revealed several intrinsic connectivity networks (ICN) playing distinct functional roles [13, 14]. Previous ICN studies demonstrate that the human brain is organised into a hierarchical, modular structure, with densely interconnected regions forming functional modules and sparse connections between modules, reflecting functional specialisation and segregation [15–17]. This organisation supports specialised processing within modules while reducing interference, thereby facilitating cognitive performance [18].

Normal ageing is associated with reorganisation of brain networks, including reduced functional specialisation and segregation, even in the absence of neurodegenerative diseases [19, 20]. Importantly, these brain changes are associated with cognitive performance decline in ageing [21–24]. Studies indicate age-related declines in the functional specialisation of default mode (DMN) and executive control networks (ECN), with reduced DMN-ECN segregation correlating with slower processing speeds [21]. Ageing also results in diminished intra-connectivity in primary processing and dorsal attention networks, which are associated with general cognitive performance [24]. Furthermore, application of graph theoretical analysis, a powerful tool to elucidate the complex network organisation at regional and system levels [25], further reveals age-related shifts towards a more integrated brain network topology [24]. Older adults exhibit lower local efficiency, indicative of reduced segregated processing and higher participation coefficients in control and dorsal attention networks, reflecting increased integrated processing [22].

Although BCI-based cognitive training shows potential for improving cognitive performance in older adults, little is known about its effects on brain network organisation. To address this gap, we aimed to investigate topological alterations in intrinsic brain functional networks following BCI-based training and their behavioural relevance in cognitively normal older adults using rs-fMRI and graph theoretical analysis. Based on previous findings of reduced functional specialisation and segregation in ageing, we hypothesised that BCI training would enhance functional segregation and specialisation, particularly in core neurocognitive and subcortical networks. Additionally, we sought to examine whether these topological changes were associated with cognitive performance improvements.

## Methods

### Participants

A single-centre, randomised-controlled trial was conducted with 227 participants aged 60–80 years old. All the participants had a Clinical Dementia Rating (CDR) [26] score of 0–0.5, Mini-Mental State Examination (MMSE) [27] score of 24 or above, Geriatric Depression Scale (GDS) [28] of 4 or below. Participants had no history of neuropsychiatric disorders, no uncorrected hearing, visual or speech impairments, no colour blindness and were not using medications such as rivastigmine, donepezil, galantamine or memantine. All participants received a detailed explanation of the study before they signed an informed consent document and underwent the study protocol.

The participants were randomly assigned to one of two groups: the intervention (INT) group or the waitlist (WL) group. The INT group underwent an 8-week BCI-based cognitive training, while the WL group followed their regular routine without any INT during this period. A subset of participants underwent rs-fMRI scan at baseline and after 8 weeks (follow-up) on a voluntary basis. The final sample for imaging study included 40 participants from the INT group and 38 participants from the WT group. After careful quality control, 28 INT participants and 28 WL participants had good structural and functional MRI data at both time points. To ensure the participants had intact cognitive function, participants with CDR = 0.5 were further excluded. A CDR score of 0.5 is generally considered indicative of subjective memory complaint and mild cognitive impairment. This resulted in a final imaging analysis sample of 22 participants (aged 66.1 ± 4.2 years, 14 females) from INT group and 24 participants (aged 65.4 ± 5.4 years, 8 females) from WL group in the imaging analyses (primary dataset; Table 1). To validate the findings, we performed the same analysis using the broader sample (28 in INT and 28 in WL) that included participants with CDR = 0.5 (Validation dataset; Appendix 1 Supplementary Table 1). The validation dataset enabled us to examine whether the main effects and patterns observed in the primary dataset were maintained when more individuals with subtle cognitive difficulties were included.

**Table 1:** Demographics, imaging information and neuropsychological assessment of the participants

			Intervention			Waitlist			*P*-value
			Pre	Post		Pre	Post		
Demographics									
Age, mean (SD), years			66.14 (4.22)			65.38 (5.40)			.60
Gender (female: male)			14:8			8:16			.04^*^
Education years			13.41 (3.13)			13.0 (4.24)			.71
MMSE			28.41 (1.74)			28.63 (1.31)			.63
CDR global Score			All 0			All 0			–
APOE4 (non-carrier: carrier)			18:4			21:3			.59
fMRI motion parameters									
No. of frames after motion scrubbing			203.87 (30.77)	194.73 (34.14)		207.88 (24.26)	202.46 (27.46)		.50
Neuropsychological assessment									
Total RBANS			101.14 (10.37)	102.50 (11.63)		101.13 (9.59)	102.42 (14.95)		.98
RBANS domain	Attention		102.14 (11.21)	107.36 (11.05)		100.67 (13.67)	102.92 (13.06)		.51
	Delayed memory		105.32 (14.57)	105.77 (14.0)		107.08 (11.66)	106.63 (12.76)		.84
	Immediate memory		88.05 (12.30)	87.55 (13.04)		88.42 (11.71)	85.50 (15.85)		.60
	Language		101.82 (15.78)	98.41 (17.60)		104.58 (12.56)	106.88 (15.59)		.14
	Visuospatial construction		109.0 (9.49)	110.23 (10.04)		105.75 (8.25)	106.33 (14.88)		.84
Total RBMT			17.64 (3.37)	19.32 (3.29)		18.33 (3.51)	18.33 (3.47)		.10

For demographics, *P*-values were derived from two-sample *t*-tests; for other scores, *P*-values represented time−group interaction effects analysed using two-way repeated ANOVA. The designator ^*^ indicates P-value < 0.05.

### BCI-based intervention procedure

**Figure 1:**
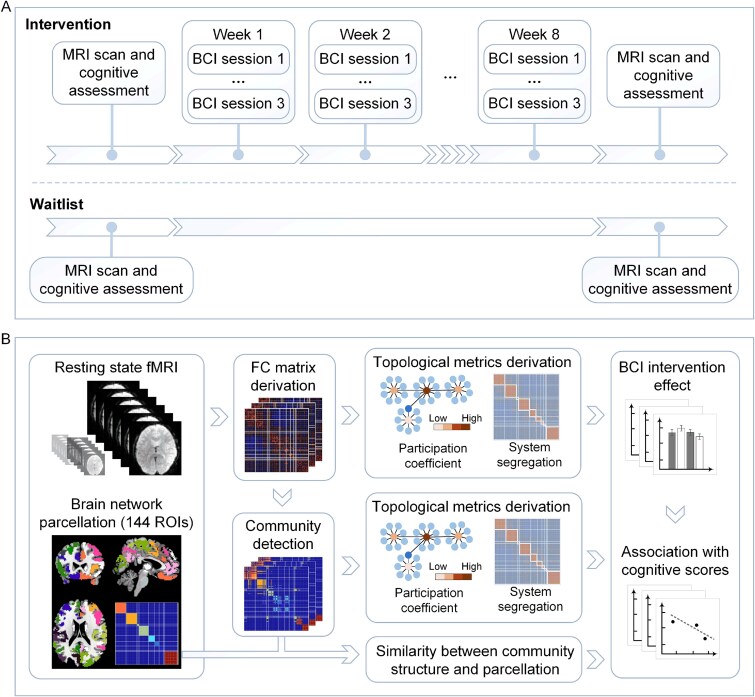
Study design schematic diagram. (A) Participants were randomly assigned to either the INT group or the WL group. Both groups completed rs-fMRI and neuropsychological assessments at baseline and follow-up. The INT group underwent an 8-week BCI-based cognitive training programme (three sessions per week), while the WL group did not receive any INT during the study period. (B) FC matrices were constructed for 144 brain ROIs at both time points. Key topological metrics, including participation coefficient and system segregation, were calculated. Community detection was applied to identify modular structures, and these were used to compute additional metrics, including the similarity of individual modular organisation to a young adult-based 144-ROI parcellation template (adjusted Rand index). These metrics were then used to examine the effect of the BCI-based INT on brain networks and brain-behavioural associations.

The participants in INT group underwent thrice-weekly training sessions for 8 weeks (Figure 1). The BCI INT was conducted using the BRAINMEM cognitive training system (see previous work [5]), where participants wore an adjustable BCI headband with dry EEG electrodes to control a computer game. Prior to the training sessions, participants completed a colour Stroop task calibration [29], which generated a personalised EEG profile used for neurofeedback during the subsequent training. Each session lasted 40 minutes and included several rounds of gameplay, consisting of three segments: shopping list, card matching and shopping list recall. These tasks targeted attention, working memory and delayed recall. As training progressed, participants practiced regulating their attention so that shopping list items appeared clearer and cards opened or closed faster. They also developed strategies to remember and recall items, cards and faces.

### Neuropsychological measurements

Neuropsychological assessments, including the Repeatable Battery for the Assessment of Neuropsychological Status (RBANS) [30] and the Rivermead Behavioural Memory Test (RBMT-II) [31], were administered at baseline and after 8 weeks. The RBANS is a well-validated and reliable cognitive screening battery that assesses attention, delayed memory, immediate memory, language and visuospatial construction. The RBMT-II detects everyday memory function by simulating real-life tasks. The efficacy outcome was defined by the change in RBANS total score, RBMT-II total scale and the five RBANS domain scores from baseline to after the INT participants completed the 8-week INT.

### Imaging acquisition and preprocessing

All the MRI images were collected at the Centre for Translational Magnetic Resonance Research, National University of Singapore, using a 20-channel head coil on a 3-T Prisma scanner (Siemens, Germany). Both resting-state functional and structural MRI images were preprocessed using a standard pipeline following our previous study [32]. Motion scrubbing was performed to minimise spurious FC in brain networks. More details on MRI parameters and preprocessing were described in Appendix 1 Supplementary Methods.

### Topological metrics and modular community detection

We extracted region-of-interest (ROI) time series using a 144-ROI rs-fMRI-based brain functional parcellation scheme [32, 33]. This parcellation was derived from 1000 healthy young adults and it allows grouping of ROIs into seven intrinsic connectivity networks (default mode network, ECN, salience/ventral attention network, dorsal attention network, somatomotor network, visual network and limbic network) and 30 subcortical regions forming a subcortical network. A FC matrix was generated for each individual at each time point using Pearson’s correlation between all pairs of ROIs, followed by Fisher’s *r*-to-*z* transformation [32].

To characterise the brain network topology, we derived graph theoretical metrics including nodal participation coefficient (which measures the diversity of a region’s connections across all systems) and global system segregation (which quantifies intersystem connectivity relative to intra-system connectivity) from individual FC matrices [19, 34] (Figure 1B). Only positive values in the FC matrices were considered, while negative values were set to zero. To make sure that results were not contingent upon the choice of a specific network density threshold, we derived the topological measures from FC matrices across a variety of network density thresholds (10%–40% with a step of 1%) and then took the integration across all thresholds for statistical analyses [32]. Participation coefficients for each node were calculated according to the following equation [34]:


(1) \begin{equation*} {P}_i=1-\sum_{m=1}^M{\left(\frac{k_i(m)}{k_i}\right)}^2, \end{equation*}

where $M$ is the number of brain systems (or modules), ${k}_i(m)$ is the weighted connections of node $i$ with nodes in system $m$ (a system to which node $i$ does not belong) and ${k}_i$ is the total weighted connections node $i$ exhibits. A high participation coefficient indicates that a node connects uniformly across multiple modules, acting as an integrative hub, whereas a low participation coefficient suggests that a node’s connections are primarily confined within its own module, reflecting functional specialisation. System segregation takes the differences in mean within-system and mean between-system correlation as a proportion of mean within-system correlation, as noted in the following formula [19]:


(2) \begin{equation*} \mathrm{system}\ \mathrm{segregation}=\frac{{\overline{Z}}_w-{\overline{Z}}_b}{{\overline{Z}}_w}, \end{equation*}

where ${\overline{Z}}_w$ is the mean within-system connectivity and ${\overline{Z}}_b$ is the mean between-system connectivity. Higher system segregation reflects stronger internal coherence within functional systems and weaker connectivity between different systems, indicating greater network specialisation and functional differentiation.

To minimise potential bias from applying a parcellation template derived from young adults, we additionally identified brain modular organisation and its INT-related changes using a fully data-driven approach, followed by the evaluation of the same topological metrics based on this data-driven modular structure. To derive data-driven brain network community structures, we used the Louvain method followed by a consensus-based clustering method [35, 36]. For each individual at each time point, modular community partition was conducted across a variety of thresholds (top 10%–40% with a step of 0.1%; 301 thresholds in total), using the Louvain method [37]. A co-classification matrix was constructed where each element represented the fraction of 301 times that a given pair of brain nodes was assigned to the same community. The consensus-based clustering method [35] was then performed on this co-classification matrix, converging to a single final consensus partition. Then we used the same steps to generate representative group-level community structures for each of the four groups (INT pre, INT post, WL pre and WL post), i.e. community detection was performed on a group-level allegiance matrix that was created by summing the consensus community assignments of all individuals in the group [36]. Community detection at both individual level and group level was performed using a range of γ parameters from 1 to 6 (resolution parameter γ determines the number of communities detected). Large values of γ bias the algorithm towards detecting smaller communities. We reported findings using individual-level γ = 3 and group-level γ = 3, as the brain network communities derived with these two parameters best reflected the normative network organisations in young adults described in previous literature [22, 33, 36].

We utilised an adjusted Rand index to quantify the alignment between individual-level community assignments and the standard 144-ROI parcellation template [36]. For validation, the same topological metrics based on the detected group-level modular structure were also calculated.

### Statistical analyses

To assess the behavioural improvements in the INT group compared to the WL group at 8 weeks, we performed a two-way repeated measures analysis of variance (ANOVA) on the RBANS total score, RBMT-II total score and RBANS domain scores.

To examine the potential group (INT vs. WL) and time (pre- vs. post-INT) interaction effects on topological metrics, we conducted two-way repeated measures ANOVA on system segregation and participation coefficient, using permutation testing (5000 permutations, α = 0.05). Age, gender and years of education were included as covariates. The false discovery rate (FDR) method was applied to correct for multiple comparisons. The same analysis was performed on the adjusted Rand index to examine changes in modular organisation.

Finally, we used Pearson’s correlation analyses to test whether changes in topological metrics were associated with cognitive improvement over time. This included changes in RBANS total score, RBMT-II total score and the RBANS domain of delayed memory and language, which were shown to respond to BCI INT in the previous behavioural-only trial [5].

## Results

### Demographics and baseline characteristics

There were no significant differences between the INT and WL groups in demographic variables, including age, years of education, MMSE scores, CDR global scores, APOE4 genotype distribution or imaging metrics (e.g. number of volumes) in the primary dataset (Table 1). The INT group included a relatively higher proportion of females compared to the WL group. Demographic details for the validation dataset are provided in Appendix 1 Supplementary Table 1.

### Behavioural changes following BCI-based intervention

After the 8-week BCI-based INT, there were no significant group-by-time interaction effects on total RBANS or RBMT scores, nor on any RBANS domain scores, within the neuroimaging subcohort (Table 1).

### Brain network reorganisation associated with BCI-based intervention

**Figure 2:**
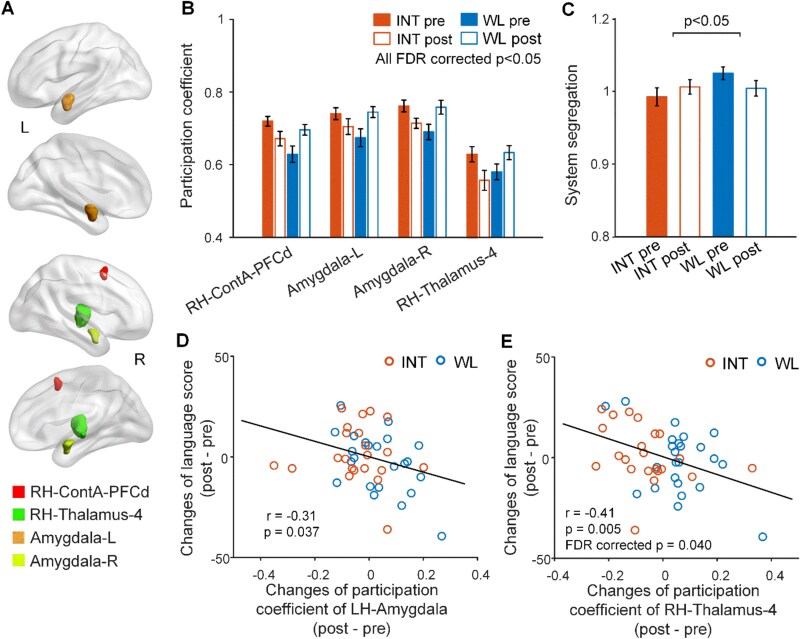
BCI-based INT in healthy older adults is associated with brain network reorganisation supporting behavioural improvement. (A and B) Brain nodes showing a significant group–time interaction effect on the participation coefficient, primarily within the ECN and subcortical networks (FDR-corrected *P* < .05). (C) Whole-brain system segregation exhibited a significant group–time interaction effect (*P* < .05). (D and E) Changes in nodal participation coefficient, particularly in subcortical regions, were significantly correlated with improvements in language performance across participants (*P* < .05). Error bars in all panels represent standard errors.

Following the BCI INT, the INT group exhibited decreased nodal participation coefficient, mainly in ECN and subcortical networks (FDR corrected *P* < .05; Figure 2A and B), along with increased system segregation compared to the WT group (*P* < .05; Figure 2C). These changes suggest enhanced network specialisation and functional segregation after the INT. Notably, participants who showed greater reductions in participation coefficients, particularly within subcortical regions, demonstrated greater improvements in language scores (*P* < .05; Figure 2D and E).

Similar results were observed in the larger dataset, which included participants with CDR global scores of 0.5 (Appendix 1 Supplementary Figure 2).

### Improved brain modular architecture with BCI-based intervention

**Figure 3:**
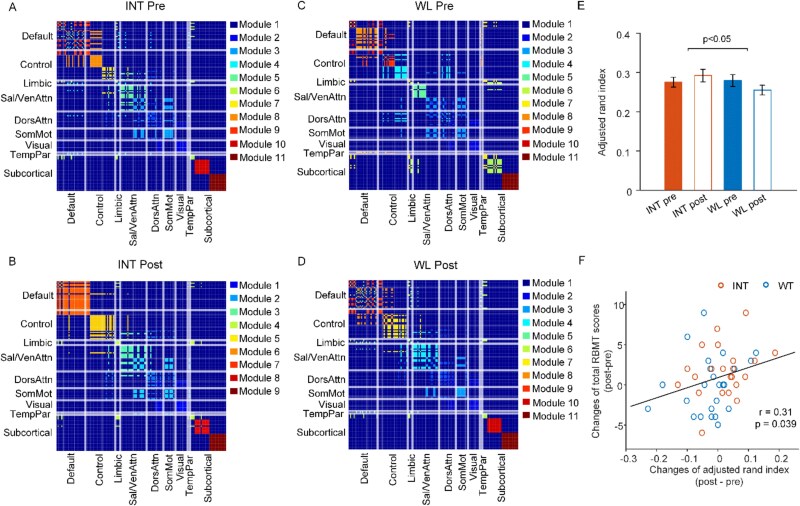
Altered network architecture following BCI-based INT in healthy older adults. Consensus matrices illustrating the modular community structures for each group and time point: (A) INT group at baseline, (B) INT group after the INT, (C) WL group at baseline and (D) WL group after the wait period. Nodes within the same community are connected by edges of the same colour, highlighting modular organisation. (E) The similarity between the detected modular architecture and the 144-region parcellation template, evaluated using the adjusted Rand index, revealed a significant group-time interaction effect (*P* < .05). Error bars represent standard errors. (F) Changes in the adjusted Rand index were positively correlated with improvements in total RBMT scores.

The modular structure of the brain functional network in the INT group became more segregated following the BCI INT (Figure 3A–D), with an increased resemblance to the young adult-based partition template, as indicated by a higher adjusted Rand index compared to the WL group (*P* < .05; Figure 3E). Specifically, both the ECN and default mode network showed increased integration in the INT group after the INT, while such change was not observed in the WL group. Greater increases in the modular structure alignment to the young adult-based partition template, as measured by the Rand index, were associated with improvements in total RBMT scores assessing everyday memory function (*r* = 0.31, *P* = .039; Figure 3F).

Key findings, including decreased nodal participation coefficients and increased system segregation, remained consistent when topological metrics were calculated using detected modular community structure rather than the standard template (Appendix 1 Supplementary Figure 1).

The larger validation dataset, which included participants with CDR global scores of 0.5, produced similar findings (Appendix 1 Supplementary Figure 3 and Supplementary Figure 4), suggesting that the observed effects were not solely dependent on the smaller, strictly defined cognitively normal imaging subgroup and may also extend to older adults with mild cognitive impairment.

## Discussion

We presented evidence for brain network reconfigurations induced by BCI-based cognitive training in cognitively normal older adults. Following the INT, although the behavioural improvements were not significant, the INT group exhibited enhanced functional specialisation and segregation of brain networks. This was reflected in increased system segregation and decreased nodal participation coefficients, particularly within the ECN and subcortical network. Additionally, the modular organisation of functional networks became more segregated and more closely resembled a young adult-based partition template. Importantly, these functional network changes correlated with cognitive performance improvements, particularly in language and everyday memory functions. These findings underscore the utility of network-sensitive neuroimaging methods in uncovering brain plasticity mechanisms associated with cognitive training in cognitively normal ageing.

Although the neuroimaging subcohort did not show significant improvements in overall cognitive performance or individual domains such as attention or delayed memory, this may be attributed to the relatively small sample size and the short duration of the INT. Notably, in the larger behavioural trial, cognitive performance gains were observed, particularly among men, in domains including delayed memory and language [5]. These findings highlight the potential sex-specific effects of the BCI system and suggest its utility for safe and acceptable cognitive training in cognitively normal older adults, especially males. It is plausible that the cognitive performance disparities between sexes may result from the interaction of historical sociodemographic factors with sex hormones, as well as differences in brain structure between men and women [5]. Recent studies have shown that cognitively normal women tend to exhibit worse resting-state functional network topology and connectivity, as well as faster rates of functional brain changes compared to cognitively normal men during ageing, which could contribute to their differing vulnerability to cognitive impairment [38, 39]. Ultimately, while there are multiple potential explanations for the sex-related effects observed in this INT, examining sex-specific brain network changes in a larger and more balanced sample is essential.

Despite the lack of significant behavioural outcomes in this study, the neuroimaging analyses revealed robust functional network reorganisation. Nodes within the executive control and subcortical networks exhibited reduced participation coefficients, indicating that connections of these nodes became more contained within their respective systems. Additionally, data-driven modular community detection showed increased integration within the ECN and default mode network (DMN), reflecting enhanced functional specialisation. The ECN and DMN are core neurocognitive networks with distinct roles; the DMN supports internally oriented mentation and autobiographical memory, while the ECN facilitates externally oriented processes requiring high cognitive demand or control [40]. Functional competition between these systems, evidenced by their anticorrelated activity, is thought to optimise cognitive performance [41, 42]. Previous studies have linked disrupted functional integration within the ECN and DMN, as well as reduced segregation between these systems, to cognitive decline [21, 22, 43]. Our findings suggest that BCI-based training may restore intersystem competition between the DMN and ECN, potentially supporting cognitive resilience in cognitively normal older adults.

The observed reduction in subcortical participation coefficients correlated with improved language scores across participants. While the effect sizes were moderate, they are comparable to those reported in prior fMRI studies on ageing [23, 44, 45]. Notably, these associations were based on longitudinal changes, which help mitigate shared variance among age, brain measures and cognitive performance, thereby offering a more specific and meaningful characterisation of brain–behaviour relationships [46, 47]. Subcortical structures such as the basal ganglia and thalamus play a crucial role in language processing [48–50], interacting with cortical regions through loops involving the prefrontal, premotor, parietal and temporal cortices [48, 51]. Task-based fMRI studies have shown that these subcortical regions are activated during language tasks [52], and their disrupted connectivity has been associated with language impairments [50]. Our findings highlight the importance of subcortical functional specialisation in maintaining language function in healthy ageing.

Normal ageing is associated with a decline in the modular and hierarchical organisation of brain networks, characterised by reduced within-network connectivity and increased between-network connectivity, as well as less system segregation and lower ‘rich club’ organisation [53]. Such changes are linked to cognitive decline [46]. In our study, participants in the INT group exhibited increased system segregation and modular similarity to a young adult-based functional parcellation template, reflecting a shift towards a more segregated and youthful brain network configuration. In contrast, participants in the WL group showed no such improvements, with some following trajectories resembling normal ageing-related network degeneration. Importantly, the modular changes were associated with improvements in everyday memory function, suggesting that BCI-based training may help mitigate or delay ageing-related brain network deterioration.

While the ageing brain inevitably experiences declining structural and functional integrity, a growing body of research shows that older adults can still benefit from lifestyle INTs such as aerobic exercise and dietary modifications [54]. Moreover, certain types of cognitive training strategies have demonstrated durable effects and strong near-transfer benefits to untrained tasks [55, 56]. For example, Requena *et al*. [55] found that a 6-year extended memory training programme significantly improved everyday memory and mental ability scores, highlighting the potential for sustained cognitive benefits through continued practice. Moreover, Grönholm-Nyman *et al*. [56] reported that a 5-week intensive set-shifting training programme led to long-lasting improvements (up to 1 year) on trained tasks. Although our study does not provide direct evidence of long-term outcomes, the observed brain network changes, particularly the increased network segregation, may reflect neuroplastic adaptations; such adaptations might help preserve cognitive function over time by supporting more efficient and specialised neural processing, thereby contributing to long-term cognitive resilience against age-related decline [57–59]. Nevertheless, future longitudinal studies are needed to test these hypotheses and to evaluate the durability of the neural changes observed in response to BCI-based INTs.

This study has several limitations. First, the sample size was relatively small after excluding participants with poor-quality data due to motion artefacts. Second, while the larger behavioural trial showed sex-specific improvements [5], we did not examine the gender effects on brain network reorganisation due to the limited sample size. The observed sex differences highlight the need for future studies to incorporate sex as a factor in analyses of brain network responses to INT. Additionally, longer-duration BCI-based cognitive training programmes and long-term follow-up assessments are needed to assess the sustainability of these effects.

In conclusion, our findings provide preliminary evidence that BCI-based cognitive training may promote healthy ageing by enhancing brain network functional segregation, with the potential to help prevent or slow network deterioration. These results offer insights into possible neural mechanisms underlying BCI-based cognitive INTs and suggest that such approaches could, with further validation, play a role in supporting cognitive function in older adults.

## Supplementary Material

aa-25-0193-File005_afaf250

## Data Availability

The raw data pertaining to this study have been kept in the database of the investigators’ group. Data access and sharing can be arranged by contacting the corresponding authors upon reasonable request. Data sharing will require a data transfer and collaborative agreement with the requestor.
