# Supplementary material for: Brain-computer-interface-based intervention increases brain functional segregation in cognitively normal older adults
Source: Age Ageing. 2025 Sep 12;54(9):afaf250. doi: 10.1093/ageing/afaf250 (PMC12445871; doi:10.1093/ageing/afaf250)
Supplement: aa-25-0193-File005_afaf250 [file aa-25-0193-file005_afaf250.docx]

**Brain-computer-interface-based intervention increases brain functional segregation in** **cognitively normal older adults**

**Appendix 1**

**Content List:**

**SUPPLEMENTARY METHODS**

**SUPPLEMENTARY TABLES**

**SUPPLEMENTARY FIGURES**

**SUPPLEMENTARY METHODS**

**Imaging acquisition**

All functional and structural MRI images were collected at the Centre for Translational Magnetic Resonance Research, National University of Singapore using a 20-channel head coil on a 3-T Prisma scanner (Siemens, Germany). The 8-min rsfMRI data comprising T2*-weighted echo planar images (repetition time = 2000 ms, echo time = 30 ms, flip angle = 90°, field of view = 192 × 192 mm2, voxel size = 3.0 mm isotropic, slice thickness = 3 mm, no gap, 36 axial slices, interleaved collection) were collected while the participants were asked to relax and stare at a cross centered on a screen. An eye tracker (ViewPoint EyeTracker, Arrington Research) was used to ensure that the participants stayed awake for the entire rsfMRI scan. The high-resolution structural T1-weighted magnetization prepared rapid gradient echo images (repetition time = 2300 ms, echo time = 2.28 ms, inversion time = 900 ms, flip angle = 8°, field of view = 256 × 256 mm2, voxel size = 1.0 mm isotropic) were collected for atlas registration of the rsfMRI images.

**Imaging preprocessing**

Both resting-state functional and structural MRI images were preprocessed using a standard pipeline based on the FMRIB's Software Library (1) (FSL, [www.fmrib.ox.ac.uk/fsl](http://www.fmrib.ox.ac.uk/fsl)) and the Analysis of Functional NeuroImages software program (AFNI) (2). The structural image preprocessing included: 1) image noise reduction, 2) skull stripping, 3) linear and none-linear registration to the Montreal Neurological Institute (MNI) 152 standard space, and 4) segmentation of the brain into gray matter, white matter and cerebrospinal fluid (CSF) compartments. For the functional images, before preprocessing, the first five volumes of each data set were discarded due to magnetic field instability at the start of data acquisition. Preprocessing steps included 1) interleaved slice-timing correction, 2) motion correction using first functional image with skull, 3) skull stripping, 4) despiking and grandmean scaling, 5) spatial smoothing using a 6 mm full width half maximum (FWHM) Gaussian kernel to improve signal-to-noise ratio and to reduce inter-subject variability, 6) temporal band-pass filtering (0.009 - 0.1 Hz) and detrending (first and second order), 7) structural MRI coregistration using Boundary Based Registration (BBR), and nonlinear registration (FNIRT) to the MNI 152 stereotactic standard space of 2 mm isotropic resolution, and 8) nuisance signals reduction by regressing out signals estimated from global signal, CSF, white matter, and six motion parameters. Registration and normalization quality was visually inspected for each session of all participants. Subsequently, we performed motion scrubbing to minimize spurious functional connectivity in brain networks. Frame displacement (FD) and the rate of change of BOLD signal across the entire brain (DVARS) (3) at each frame were calculated and frames with FD larger than 0.2 and DVARS larger than 0.005 were removed.

**SUPPLEMENTARY TABLES**

**Supplementary Table 1. Demographics, imaging information, and neuropsychological assessment of the participants from validation dataset**

|  | |  | **Intervention (N=28)** | |  | **Waitlist (N=28)** | |  | P-value |
| --- | --- | --- | --- | --- | --- | --- | --- | --- | --- |
|  |  |  | **Pre** | **Post** |  | **Pre** | **Post** |  |  |
| ***Demographics*** | | | | | | | | | |
| Age, mean (SD), years | |  | 65.86 (4.31) | |  | 65.54 (5.21) | |  | 0.80 |
| Gender (female : male) | |  | 17:11 | |  | 11:17 | |  | 0.11 |
| Education years | |  | 13.29 (3.04) | |  | 12.61 (4.23) | |  | 0.49 |
| MMSE | |  | 28.18 (1.66) | |  | 28.40 (1.57) | |  | 0.62 |
| CDR global Score (0 : 0.5) | |  | 22:6 | |  | 24:4 | |  | 0.49 |
| APOE4 (non-carrier : carrier) | |  | 23:5 | |  | 25:3 | |  | 0.45 |
| ***fMRI motion parameters*** | | | | | | | | | |
| No. of frames after motion scrubbing | |  | 206.04  (28.96) | 196.61  (33.69) |  | 205.36  (26.87) | 200.79  (29.75) |  | 0.61 |
| ***Neuropsychological assessment*** | | | | | | | | | |
| Total RBANS | |  | 102.21 (10.67) | 102.21 (10.61) |  | 99.50 (11.71) | 100.57 (14.89) |  | 0.73 |
| RBANS  domain | Attention |  | 102.07 (10.37) | 105.32 (11.58) |  | 100.36 (13.50) | 101.96 (12.62) |  | 0.67 |
|  | Delayed Memory |  | 106.82 (13.82) | 106.96 (14.41) |  | 106.25 (11.87) | 103.82 (13.85) |  | 0.52 |
|  | Immediate memory |  | 88.36 (13.07) | 86.68 (12.77) |  | 86.32 (14.10) | 85.64 (16.08) |  | 0.82 |
|  | Language |  | 102.68 (15.83) | 100.11 (17.31) |  | 102.82 (14.48) | 104.14 (16.26) |  | 0.30 |
|  | Visuospatial Construction |  | 110.0 (9.21) | 109.93 (9.13) |  | 104.46 (9.17) | 106.0 (14.52) |  | 0.58 |
| Total RBMT | |  | 18.04 (3.23) | 19.36 (3.14) |  | 18.11 (3.36) | 18.14 (3.89) |  | 0.19 |

Note: For demographics, p-values were derived from 2-sample t-tests; for other scores, p-values represented time-group interaction effects analyzed using 2-way repeated ANOVA.

**SUPPLEMENTARY FIGURES**





**Supplementary Figure 1.** **BCI-based intervention in cognitively normal older adults is associated with brain network reorganization underlying behavioral improvement (primary dataset; graph theoretical measures were calculated based on modular organization derived from community detection).** A. Nodes showing significant time and group interaction effect on participation coefficient are presented (FDR corrected p < 0.05). B. whole-brain segregation coefficient showed significant time and group interaction effect (p < 0.05). Error bars represent standard errors. Changes of nodal participation coefficient by the BCI-based intervention were correlated with the behavior improvement of language and total RBMT score in cognitively normal older adults (C, D).



**Supplementary Figure 2. BCI-based intervention in** **older adults is associated with brain network reorganization underlying behavioral improvement (validation dataset).** A. Nodes showing significant time and group interaction effect on participation coefficient are presented (FDR corrected p < 0.05). B. whole-brain segregation coefficient showed significant time and group interaction effect (p < 0.05). Error bars represent standard errors. Changes of nodal participation coefficient by the BCI-based intervention were correlated with the behavior improvement of language and total RBMT score in older adults (C, D).





**Supplementary Figure 3. Altered network architecture in older adults (validation dataset)**. Consensus matrices showing community structures in each of the 4 groups (A. Intervention pre, B. Intervention post, C. Waitlist pre and D. Waitlist post) were presented. The edges connecting nodes in the same community are highlighted using the same color. E. Similarity between detected modular architecture and 144-ROI parcellation template evaluated by adjusted rand index showed significant time and group interaction effect (p < 0.05). Error bars represent standard errors.

**

**

**Supplementary Figure 4.** **BCI-based intervention in older adults is associated with brain network reorganization underlying behavioral improvement (validation dataset; graph theoretical measures were calculated based on modular organization derived from community detection).** A. Nodes showing significant time and group interaction effect on participation coefficient are presented (FDR corrected p < 0.05). B. whole-brain segregation coefficient showed significant time and group interaction effect (p < 0.05). Error bars represent standard errors. Changes of nodal participation coefficient by the BCI-based intervention were correlated with the behavior improvement of language and total RBMT score in older adults (C, D).

**References**

1. Smith SM, Jenkinson M, Woolrich MW, Beckmann CF, Behrens TE, Johansen-Berg H, et al. Advances in functional and structural MR image analysis and implementation as FSL. Neuroimage. 2004;23:S208-S19.

2. Cox RW. AFNI: software for analysis and visualization of functional magnetic resonance neuroimages. Computers and Biomedical research. 1996;29(3):162-73.

3. Power JD, Barnes KA, Snyder AZ, Schlaggar BL, Petersen SE. Spurious but systematic correlations in functional connectivity MRI networks arise from subject motion. Neuroimage. 2012;59(3):2142-54.
